# Point-of-Care Ultrasonography Saves the Day in Dilated Cardiomyopathy: A Rare Presentation of Hyperhomocysteinemia

**DOI:** 10.7759/cureus.16699

**Published:** 2021-07-28

**Authors:** Mack Sheraton, Dhaval Patel, Richard Houck

**Affiliations:** 1 Emergency Medicine, Trinity West Medical Center, Steubenville, USA; 2 Internal Medicine, Trinity West Medical Center, Steubenville, USA

**Keywords:** dilated cardiomyopathy, hyperhomocysteinemia, intraventricular thrombus, subclavian thrombus, systolic heart failure

## Abstract

Here, we report a case of hereditary hyperhomocysteinemia presenting as dilated cardiomyopathy which was successfully diagnosed using a combination of point-of-care ultrasonography (POCUS) and echocardiogram (ECHO). A 39-year-old Caucasian male with a family history of homocystinuria and early deaths in adult male members from cardiovascular disease presented with complaints of purplish discoloration and 4/10 pain in bilateral feet along with severe nausea/vomiting for the last two days. Physical examination was significant for tachycardia, low normal mean arterial pressures, dry mucous membranes, right basilar crepitations, S3 gallop with holosystolic murmur along with peripheral cyanosis, and pitting edema. Laboratory examination revealed leucocytosis, elevated d-dimers, high anion gap metabolic acidosis secondary to worsening renal function, elevated liver enzymes, hyperhomocysteinemia, elevated B-type natriuretic peptide, and troponins along with low protein C and S. Electrocardiogram demonstrated left axis deviation with abnormal QRS-T angle and intraventricular conduction delay with a QRS duration of 133 ms. Bedside POCUS and ECHO revealed marked left ventricular dilatation with an ejection fraction of 10% and mitral regurgitation. Computed tomography angiography of the chest and abdomen was positive for partial left subclavian vein thrombus with extensive collateral formation and right-sided pleural effusion. The patient was started on anticoagulants and promptly transferred to a tertiary care center for left ventricular assist device placement. Hyperhomocysteinemia can present with atypical heart failure symptoms, and early usage of bedside POCUS and interpretation of findings in the context of family history are imperative for a successful diagnosis.

## Introduction

Homocysteine is a nonessential amino acid produced during the metabolism of an essential amino acid methionine. Hyperhomocysteinemia is a multisystemic metabolic disorder caused by the accumulation of homocysteine due to a failure of metabolism. Metabolism primarily occurs by transsulfuration by cystathionine β-synthase (CBS) or remethylation by methionine synthase [1]. Abnormalities in these enzymes and deficiencies in cofactors vitamin B6, vitamin B12, and folic acid required in the metabolic pathways can result in hyperhomocysteinemia [1]. One of the more severe forms is homocystinuria, an autosomal recessive disorder with a prevalence of 1:150,000 live births worldwide [2]. A genetic deficiency of CBS causes very high homocysteine levels in the blood and urine. This leads to vascular damage and atherothrombotic diseases occurring early in life due to a general prothrombotic state [3]. Patients usually present with ectopia lentis, mental retardation, developmental retardation, early thromboembolic disorders, marfanoid characteristics, spinal osteoporosis, and behavioral problems [4]. We report a case of homocysteinemia which is unique because of the unusual nature of its presentation.

## Case presentation

A 39-year-old Caucasian male with a prior history of hemorrhoidectomies, dyslipidemia, and depression presented with severe nausea, vomiting, and purplish discoloration of bilateral feet to our rural community hospital. He stated that nausea and vomiting had started about 48 hours previously. There have been no accompanying diarrhea or fever symptoms. Twenty-four hours prior to presentation, the patient noticed purplish discoloration of bilateral feet, which was slowly worsening. Initially, he had foot pain with an intensity of four on a ten-point scale, which was nonradiating and without any identifiable precipitating, aggravating, or alleviating factors. However, this was subsequently replaced with numbness and swelling. He recalled episodic chest pain and shortness of breath for the last two months, for which he had never sought treatment. These were accompanied by weakness and fatigue, which had become a fairly consistent symptom. He denied any chest pain or dyspnea at the time of presentation. The patient stated that both his father and grandfather had been diagnosed with homocystinuria. Even though they had died earlier in life, at around 30 years of age, he had never been tested for homocystinuria. He smoked about half a pack a day and had a history of alcoholism but had been sober for the last 10 years. Scrutiny of prior visits showed the primary complaint to be musculoskeletal pain from the cervical spine early on and right knee secondary to osteoarthritis more recently. Recently, he had been diagnosed with dyslipidemia, which was being treated with lifestyle modifications. He was taking olanzapine (10 mg daily) and escitalopram (20 mg daily) for depression.

Physical examination was significant for tachycardia and normal blood pressure with progressively declining mean arterial pressures ranging between 70 and 80 mmHg but normal body temperature and oxygen saturation at room air. He did have a flat affect, and his mucous membranes appeared dry. The respiratory examination was significant for mild right-sided basal crepitations, and cardiovascular examination revealed an S3 gallop associated with a holosystolic murmur best heard at the apex with the patient in the left decubitus position. Examination of extremities was significant for peripheral cyanosis of both lower extremities extending to the ankles along with one plus pitting edema. Neurological and abdominal examinations were benign.

Investigations

**Figure 1:**
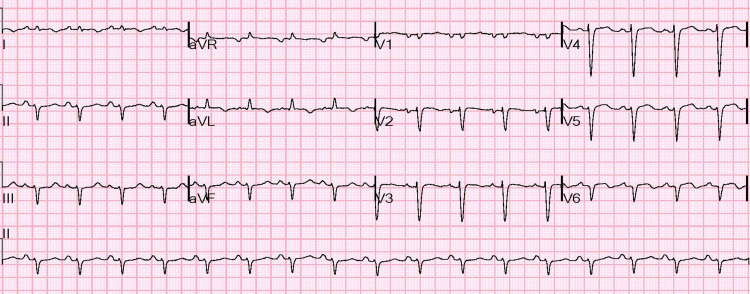
Electrocardiogram at presentation.

An electrocardiogram (ECG) done in the Emergency Department (ED) showed tachycardia with a regular rhythm and intervals, predominant left axis deviation, borderline left atrial enlargement, abnormal QRS-T angle, moderate intraventricular conduction delay due to QRS duration of 113 ms, and nonspecific ST elevations in leads V2 to V6 without any reciprocal depressions (Figure 1). Laboratory testing was significant for leucocytosis, with neutrophilia and lymphopenia, mild homocysteinemia, pre-renal azotemia with a high blood urea nitrogen/creatinine ratio and low glomerular filtration rate, elevated hepatic transaminases, and elevated total serum bilirubin. Elevated levels of quantitative d-dimer, serum troponin, and B-type natriuretic peptide (BNP) were also reported. Table 1 lists the laboratory values and their normal ranges with abnormal values highlighted.

**Table 1:** Summary of laboratory test results. WBC: white blood cell; RBC: red blood cell; Hgb: hemoglobin; Hct: hematocrit; BNP: B-type natriuretic peptide; PT: prothrombin time; INR: international normalized ratio; ESR: erythrocyte sedimentation rate; CRP: C-reactive protein; BUN: blood urea nitrogen; EGFR: estimated glomerular filtration rate; AST: aspartate transaminase; ALT: alanine transaminase; ALP: alkaline phosphatase

Laboratory findings	Values (normal range)	Laboratory findings	Values (normal range)
WBC	16.2 × 10^3^ uL (4.5-11 × 10^3^ uL)	Sodium	131 mmol/L (136-145 mmol/L)
RBC	4.35 × 10^6^ uL (4.5-5.90 × 106 uL)	Potassium	3.9 mmol/L (3.5-5.1 mmol/L)
Hgb	13.1 g/dL (13-16 g/dL)	Chloride	97 mmol/L (98-107 mmol/L)
Hct	38.6% (41-53%)	Bicarb	21 mmol/L (21-32 mmol/L)
Platelet	113 × 10^3^ uL (150-350 × 10^3^ uL)	Anion gap	16.9 (0-16)
Neutrophils	83.7% (40-80%)	BUN	60 mg/dL (7-18 mg/dL)
Lymphocytes	10.0% (13-17%)	Creatinine	1.89 mg/dL (0.7-1.3 mg/dL)
Monocytes	6.1% (0.0-10%)	EGFR	40 (>60)
Eosinophils	0.0% (0.0-10%)	BUN\creatinine ratio	31.7 (0-30)
Basophils	0.2% (0.0-1.5%)	Glucose	96 mg/dL (70-110 mg/dL)
Troponin	2.25 ng/mL (<0.05 ng/mL)	Calcium	8.5 mg/dL (8.5-10.1 mg/dL)
BNP	2,929.13 pg/mL (2-100 pg/mL)	Total bilirubin	2.4 (0.0-1.0 mg/dL)
PT	20 s (9.9-11.6 s)	AST	1,629 U/L (15-37 U/L)
INR	1.5	ALT	2,678 U/L (13-61 U/L)
PTT	28 s (22-33 s)	ALP	120 U/L (13-61 U/L)
Thrombin time	32.8 s (14.7-19.5 s)	Total protein	7.3 g/dL (45-117 g/dL)
ESR	14 mm/h (0-20 mm/h)	Lipase	386 U/L (73-393 U/L)
CRP	1.3 mg/L (0-3 mg/L)	D-dimer	>35.2 mg/L (<0.5 mg/L)
Hepatitis panel	Negative	Homocysteine	19.8 µmol/L (3.2-10.7 µmol/L)
Alcohol	Negative	Urine drug screen	Negative

**Figure 2:**
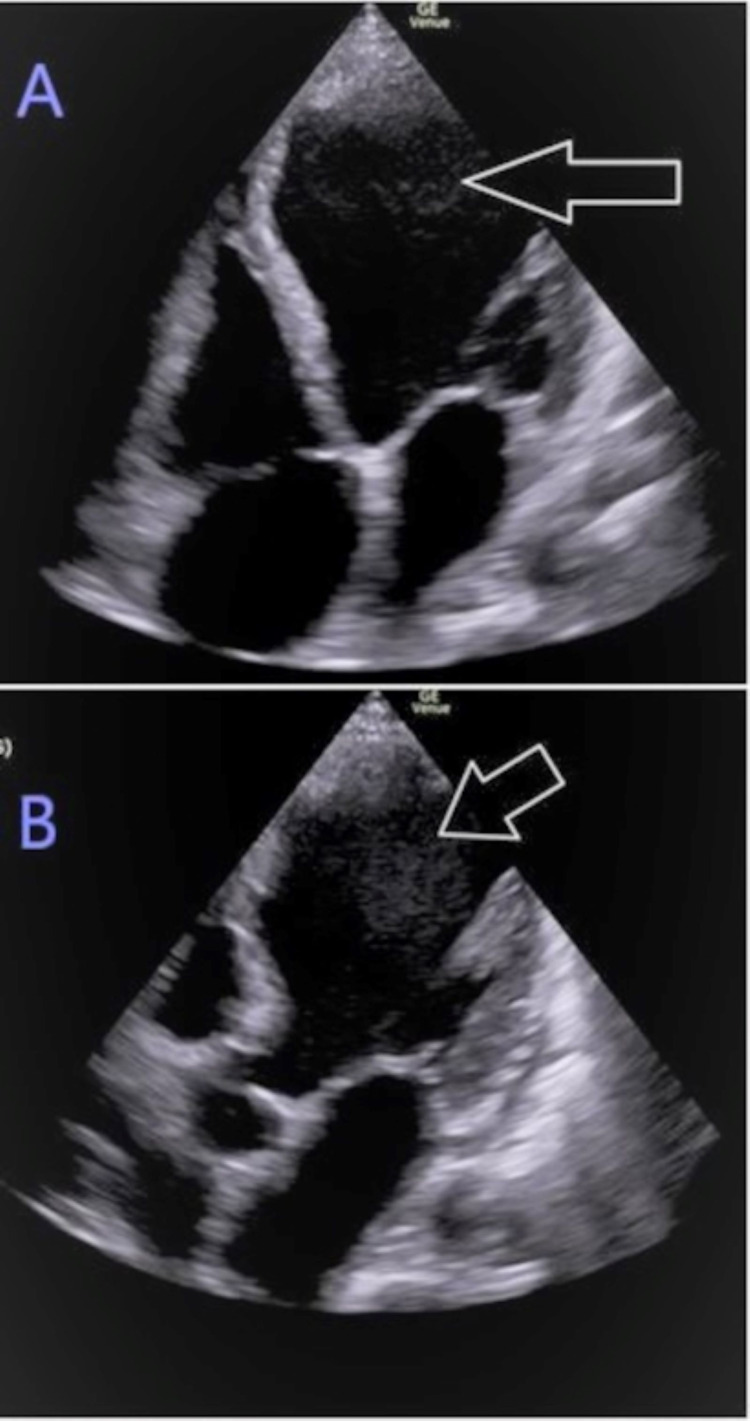
POCUS ECHO showing dilated cardiomyopathy and intraventricular thrombus (arrow). (A) apical four-chamber. (B) Apical five-chamber views. POCUS: point-of-care; ECHO: echocardiography

**Figure 3:**
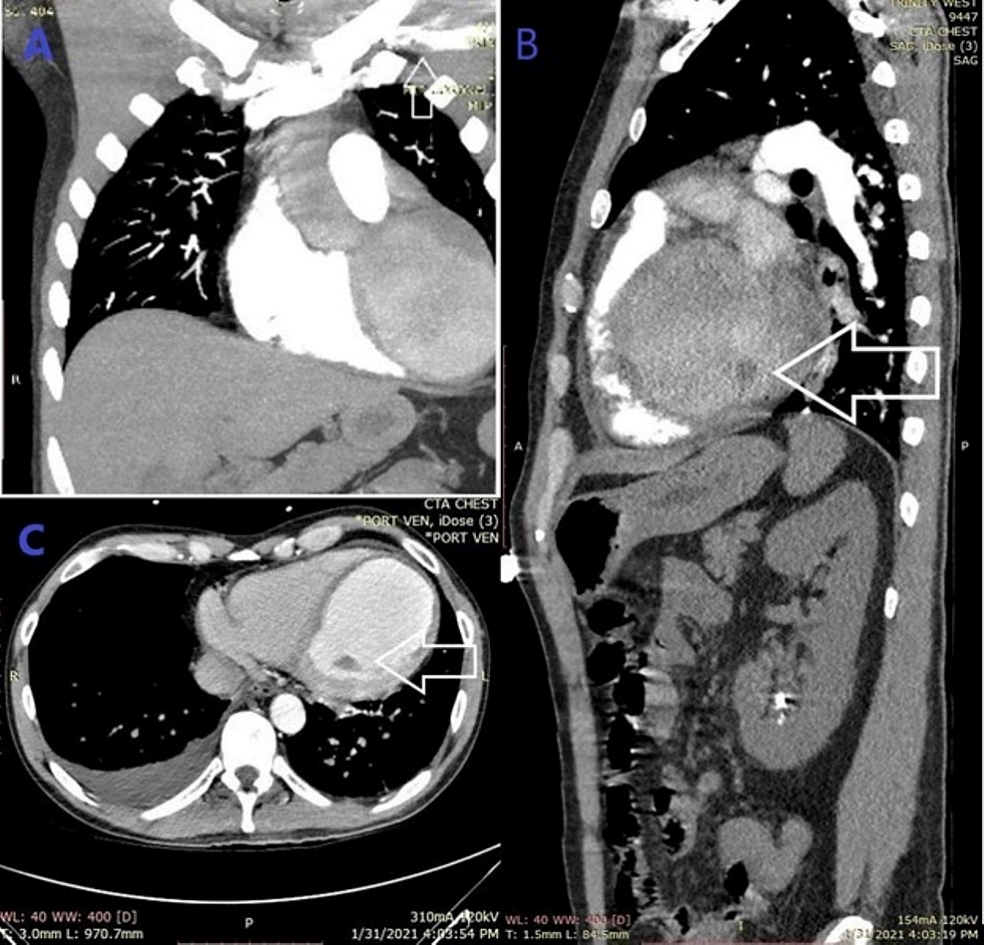
CTA of the chest. (A) Coronal view with partially occluding left subclavian vein thrombus. (B) Sagittal view with intraventricular filling defect. (C) Axial view with intraventricular filling defect CTA: computed tomography angiography

**Figure 4:**
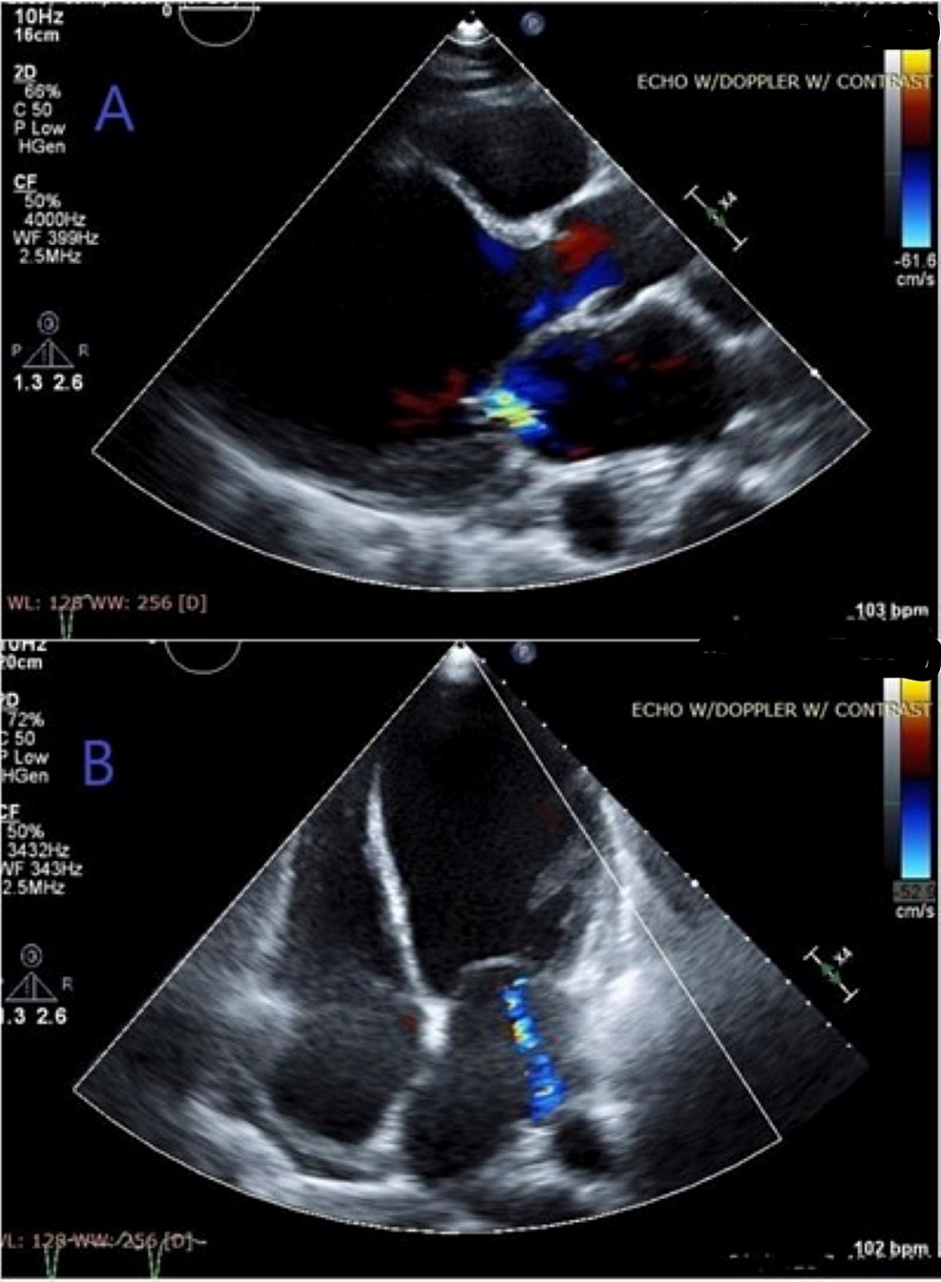
Transthoracic echocardiogram demonstrating mitral regurgitation (arrow) and dilated left ventricle in (A) parasternal long-axis view and (B) apical four-chamber view.

A bedside point-of-care (POCUS) echocardiography (ECHO) (Figure 2) showed left ventricular dilated cardiomyopathy (DCM) with an intraventricular thrombus and global hypokinesis in the apical four-chamber view and mitral regurgitation evident on parasternal long-axis view with color Doppler. Bedside limited bilateral lower extremity venous Dopplers were negative for deep vein thrombosis (DVT) but pulsed-wave Dopplers showed diminished flow in bilateral dorsalis pedis artery. Bedside limited abdominal POCUS revealed pericholecystic fluid with sludge but a negative sonographic Murphy’s sign. A subsequent computed tomography angiography (CTA) of the chest and abdomen was negative for pulmonary embolism, coronary lesions, or abdominal aortic branch occlusion but revealed partial thrombus in the subclavian vein with multiple collateral vessels in the left lower neck and upper back. There was a minor filling defect in the left ventricle suspicious for a thrombus (Figure 3). A formal two-dimensional ECHO with color Doppler confirmed POCUS findings and quantified severely diminished systolic function with an ejection fraction at 10% along with left ventricular wall thinning (Figure 4). An attempt to view the intraventricular thrombus with contrast was inconclusive because the examination was suboptimal due to a marked swirling artifact.

Differential diagnosis and treatment

At presentation, initial stabilization of the patient was started with a working diagnosis of gastroenteritis because of the presenting symptoms of nausea/vomiting. Therefore, a pharmacologic therapeutic intervention was initiated with intravenous administration of a liter of normal saline bolus and ondansetron (4 mg). The limited laboratory tests that had been drawn earlier in the ED waiting room demonstrated leucocytosis and acute renal failure, which made us consider sepsis. As a result, piperacillin/tazobactam, a broad-spectrum antibiotic, was started after drawing cultures while we awaited abdominal imaging. Homocysteine levels, cardiac markers, and D-dimer were sent after obtaining a family history of homocystinuria and bedside POCUS, which demonstrated systolic dysfunction with DCM and intraventricular thrombus. Simultaneous findings of hyperhomocysteinemia, decompensated cardiac functions, and elevated dimers made us revisit our working diagnosis. Predominant etiologies for the DCM that we considered at this point included familial, infections (viral cardiomyopathy), connective tissue diseases, and toxin-induced (alcohol, other drugs of abuse). History was negative for a prior diagnosis of any connective tissue disorder, recent alcoholism, or abuse of illicit substances. Erythrocyte sedimentation rate and C-reactive protein levels were within the normal range. Similarly, serum alcohol and urine drug screens were negative. However, viral titers, specifically enterovirus-specific antibody titers, were sent but results were not obtained before the transfer. Nonetheless, when the picture of a hypercoagulable state with heart failure evolved, antibiotics were stopped, and heparin 6,000 IU bolus followed by drip titrated to activated partial thromboplastin time was started. CTA of the chest and abdomen demonstrating intraventricular and partial subclavian vein thrombus confirmed our suspicions. We derived that increased liver enzymes, compromised renal function, pericholecystic fluid, elevated troponins, and BNP were all a function of compromised cardiac function. We consulted the cardiology service for contrast ECHO, and after obtaining the results, they advised transfer to a tertiary care facility for a left ventricular assistive device placement. Because of the lack of severe symptoms with such a low systolic function, we concluded that this was a chronically progressive process aggravated suddenly by hypovolemic stress secondary to vomiting. The patient was given 0.5 mg of hydromorphone for symptomatic pain relief and transferred by critical care air transport.

Outcome and follow-up

At the tertiary care center, the patient received a left ventricular assist device immediately with dietary treatment. He started having frequent episodes of ventricular tachycardia and ventricular fibrillation (VT/VF), for which he received an implantable cardio defibrillator. Viral titers and blood cultures that had been drawn in the ED were negative after 48 hours. Investigations for possible alternative causes for DCM done at the tertiary care center were all negative. Afterward, when he continued to have VT/VF episodes, he was deemed a candidate for and soon received a heart transplant. Subsequently, the patient was discharged to inpatient rehabilitation after spending 37 days in the hospital. He is continuing with outpatient cardiac rehabilitation and follows up with the transplant team to this day.

## Discussion

We report a case of DCM in a patient with a family history of homocystinuria. To our knowledge, DCM with atypical heart failure features has never been described as a presenting finding in a hyperhomocysteinemia patient. The primary strength of our approach to the diagnosis was to access cardiovascular function with early deployment of bedside POCUS. Early findings of a dilated ventricle with mitral regurgitation and intraventricular thrombus helped us change therapeutic intervention to anticoagulation at the right time. Enlisting cardiology consultation early on helped in the proper triaging of the patient to a tertiary care center.

Very commonly, left ventricular systolic dysfunction (LVSD) progresses through an asymptomatic period before becoming apparent. The most common causes of LVSD are coronary ischemic heart disease (IHD), idiopathic dilated cardiomyopathy (DCM), hypertension, and valvular disease [5]. In our case, IHD was ruled out because of lack of symptoms and ECHO findings not demonstrating regional abnormalities of left ventricular wall motion and/or thickening during systole. Though mitral regurgitation was detected, it was of such a small degree that we attributed it to being functional due to DCM rather than the primary cause of LVSD. Studies conducted on those presenting initially with unexplained DCM found the most frequent causes to be idiopathic (50%), myocarditis (9%), IHD (7%), and infiltrative diseases (5%) [6]. Hyperhomocysteinemia has been found to have a significant association with heart failure. Recent studies conducted on the genomic level have indicated that it is a causal factor for nonischemic heart failure in DCM [7].

A review of contemporary medical literature suggests that the pathological basis for the cardiovascular effects of hyperhomocysteinemia is an injury to the arterial endothelium and smooth muscles resulting in vascular wall remodeling through a fibrous luminal narrowing and arterial stiffening. Secondarily, lipid deposition in these arterial lesions results in atherosclerotic arteriosclerosis [3]. A hypercoagulable state caused by oxidative stress, platelet activation, and higher prothrombotic factors has been described [8]. In addition, renal arterial pathology causing decreased elimination of homocysteine creates a self-perpetuation of cardiovascular risk [9]. Researchers have also reported fibrillin-1 protein modification causing alterations in connective tissues resulting in structural damages such as cardiac valvulopathies along with ocular and skeletal malformations, as seen in Marfan’s syndrome [10]. This might have been the basis of the DCM seen in our case.

A range of cardiovascular complications has been shown to have a solid epidemiological association with hyperhomocysteinemia, even though causality has not been wholly worked out [11]. These primarily include hypertension, coronary artery diseases, coronary dissection causing myocardial infarction, deep vein thromboses of lower extremities, ischemic stroke, cerebral venous thrombosis, and pulmonary vein thrombosis [12]. The echocardiographic abnormalities described include tricuspid regurgitation, mitral valve prolapse, left atrial enlargement, aortic valve sclerosis, mitral regurgitation, and aortic root ectasia. Similarly, the associated electrocardiographic abnormalities include left axis deviation, left bundle branch block, right bundle branch block, and early ventricular repolarization [13]. Case reports have also described presentations with postural orthostatic tachycardia syndrome, calcified right atrial mass, and isolated aortic root dilatation [14-16]. Such a range of possible abnormalities makes POCUS an indispensable tool in the early diagnosis and management of hyperhomocysteinemia patients.

## Conclusions

This case report showcases a rare condition of hereditary hyperhomocysteinemia presenting with hitherto unreported complications of dilated cardiomyopathy and intraventricular and subclavian vein thrombi. Our experience with the diagnosis of this case makes the early usage of POCUS imperative in all presentations of congenital abnormalities with known cardiovascular associations. ED physicians should prioritize bedside POCUS ECHO in suspected cases of LVSD, especially when considering DCM. Incorporating family history during the initial assessment is crucial for early and correct diagnosis. Hereditary hyperhomocysteinemia can present with dilated cardiomyopathy.
